# Sub-wavelength annular-slit-assisted superoscillatory lens for longitudinally-polarized super-resolution focusing

**DOI:** 10.1038/s41598-019-56810-3

**Published:** 2020-01-28

**Authors:** Hyuntai Kim, Edward T. F. Rogers

**Affiliations:** 1 Electrical and Electronic Convergence Department, Hongik University, Sejong, 30016 Republic of Korea; 2 Optoelectronics Research Centre, University of Southampton, Southampton, SO17 1BJ UK; 3 Institute for Life Sciences, University of Southampton, Southampton, SO17 1BJ UK

**Keywords:** Optics and photonics, Optical materials and structures

## Abstract

A binary metallic superoscillatory lens assisted with annular subwavelength slits is proposed, which generates a longitudinally-polarized super-resolution focal point. The annular slits are designed to selectively transmit radially-polarized light. Simulations using the finite element method show a 0.24 λ focal spot with 21.8 dB of polarization purity and only 0.342 dB reduction in efficiency compared to a standard superoscillatory lens.

## Introduction

The classical restriction on the resolution of focusing, caused by the diffractive nature of light and known as Abbe’s diffraction limit, has historically been a strong constraint on the resolution of various optical systems. In recent years, however, it has been shown that light can be focused well below the diffraction limit in the far field by exploiting the phenomenon of superoscillation, where light oscillates faster than its highest Fourier component in a specific local region^1–4^. Optical superoscillations have been realized by means of spatial light modulators, optical eigenmode methods, metasurfaces, and binary lenses^5–10^.

Especially important is the binary superoscillatory lens (SOL) as it is relatively easy to fabricate and has a simple structure. Various SOLs have been studied for purposes including generation of optical needles, achromatic super-resolution (SR) focusing, and far-field SR focusing^11–16^.

Longitudinally polarized (LP) light is generated whenever radially polarized (RP) light is focused down with high numerical aperture^17–21^. A true LP focus contains only polarization components in the direction of propagation, which has advantages in various applications (compared to linearly- and circularly-polarized light) due to smaller spots formed at a LP focus and radially symmetric optical forces. LP foci also improve SR focusing, thus LP-SR focusing is an essential tool in nano-applications such as particle trapping, particle acceleration, and high-resolution imaging^22,23^. Recently, sub-diffraction longitudinally polarized focusing using binary phase lenses and RP input has been reported^24,25^.

Despite these many benefits, LP foci are not widely used in practical applications, primarily because the necessary RP input is difficult to generate and align. For linearly- or circularly-polarized inputs, additional free-space optical components are required to convert the polarization state, which complicates the whole system^26^. For waveguide inputs, such as optical fibre modes, one can prepare cylindrical vector states – which are composed of a superposition of radial, azimuthal, and hybridized degenerate modes – by means of donut shaped active fibre, or long-period-grating mode couplers^27–29^. In these cases, however, an additional polarizer is still required to extract the RP component. Even after the RP input is properly prepared, the incident RP light must be carefully centered on the SOL or other focusing optic, which requires additional effort. Any misalignment of either the free-space RP generator or the focusing optic, which can easily occur due to external drifts, causes rapid degradation of modal purity which in turn degrades the LP-SR focusing effects and results in larger focal spots and/or generation of other polarization components at the focus.

It is well known that metallic subwavelength slit arrays are polarization selective: they transmit light polarized perpendicular to the slits and block the component polarized parallel to the slits. The effect can be understood by considering the movement of electrons, extraordinary optical transmission phenomena based on surface plasmon polaritons, or from effective medium theory (EMT)^30,31^. One of the most widely used applications is the wire-grid polarizer, and it is also known that annular subwavelength metallic slits can transmit RP components but block azimuthally polarized (AP) components. The authors have recently studied annular subwavelength slits theoretically and proposed a zone plate consisting of subwavelength rings which could selectively focus RP light^30^.

Here, we propose a novel sub-wavelength annular slit (SAS) assisted SOL (SAS-SOL) structure which only transmits RP light and therefore generates a LP-SR focal point, while blocking other polarization components. Inspired by metallic subwavelength ring arrays, we apply an annular substructure to SOL and achieve pure RP components which are focused to a LP-SR hotspot from a single binary metallic surface. We stress here that the method we are developing is generally applicable and can be applied to any existing SOL design. In the main body of the work below, we chose an existing design [ref. ^12^] and modify it by adding subwavelength slits for polarization selectivity. The precise lens design is not important, as is demonstrated in subsection “Robustness and general applicability” where we simulate a range of other SOL designs from the literature and demonstrate that the SAS-SOL principle works equally well with all of them.

**Figure 1:**
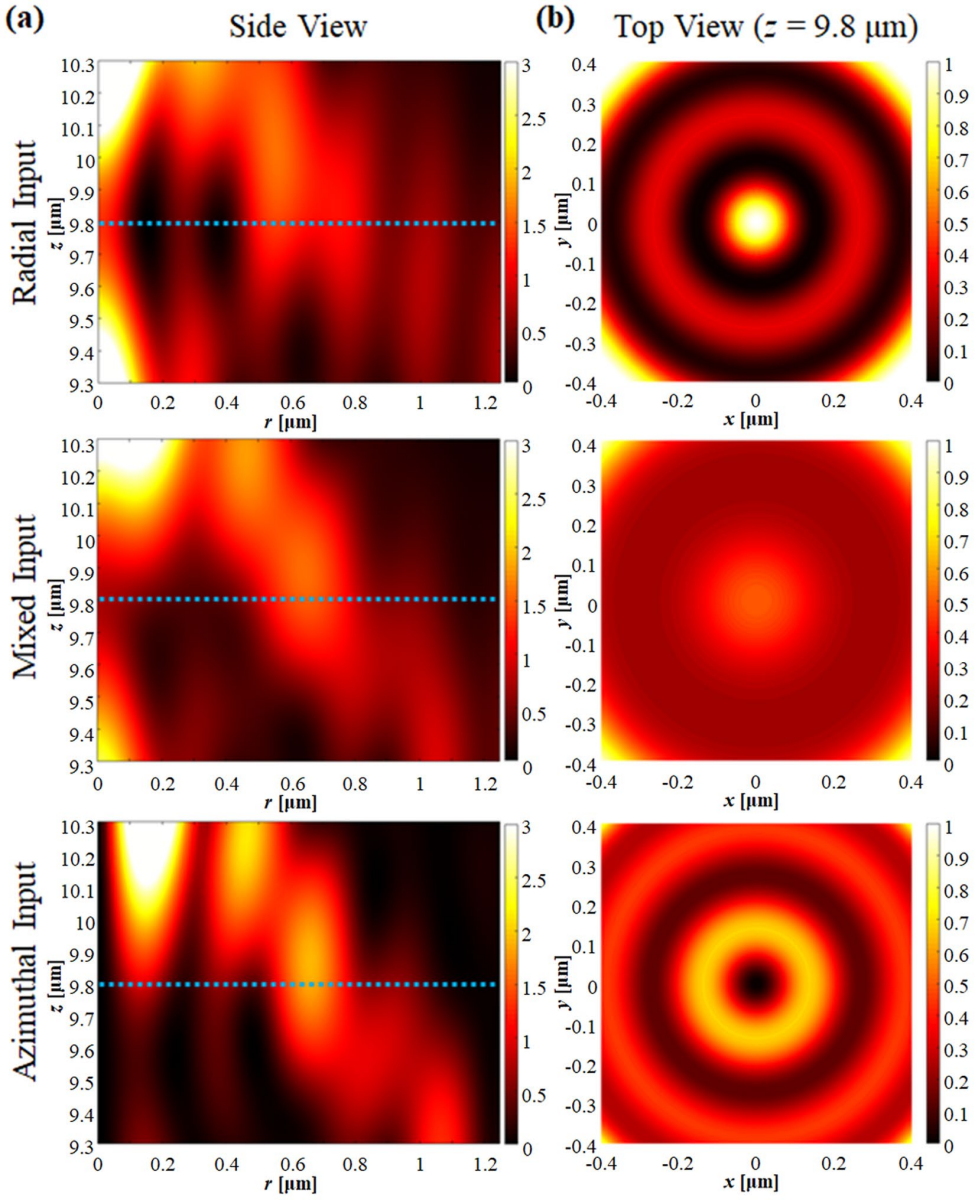
(**a**) Side view and (**b**) top view of the electric field intensity when RP, mixed, and AP light illuminates the SOL.

The most obvious light source for SAS-SOL would be mode-selected cylindrical waveguides, for example optical fibre, which generate both RP and AP components^26^. If a free-space focusing system is preferred, then prepared RP beam would be focused correctly by the SAS-SOL. It should also be noted that having the focusing component fixed on the fibre facet makes alignment much simpler and improves efficiency^6,18^. Thus, the proposed SAS-SOL simplifies the system by polarizing the input itself, and thus makes alignment significantly easier. Below, we first characterize the polarization performance of the SOL and SAS separately, and then rigorous simulations are performed to characterize and optimize the SAS-SOL to achieve high efficiency LP-SR focusing.

## Results

### SOL without subwavelength rings

To understand what is required of the SAS-SOL, we must first analyze how each polarization component is focused by the SOL. Here we use the SOL design from ref. ^12^ as the basis for our calculations. The lens has a diameter of 20 μm, and consists of 25 transparent annular regions. The full lens specification can be found in Supplementary Information of ref. ^12^. The lens which has a known focal length of 9.8 µm for 640 nm light in the immersion oil used here (see Methods). We calculate the field intensity at the SOL focus for varying ratios of RP to AP input. The focal spot (in the *xy* plane) and cross-sectional (*xz*) view of the results are shown in Fig. 1. The mixed input has equal proportion of RP and AP input. It is worth mentioning that our finite element method (FEM) simulation results show good agreement with to the previous experiment^12^, which confirms the reliability of our numerical results.

**Figure 2:**
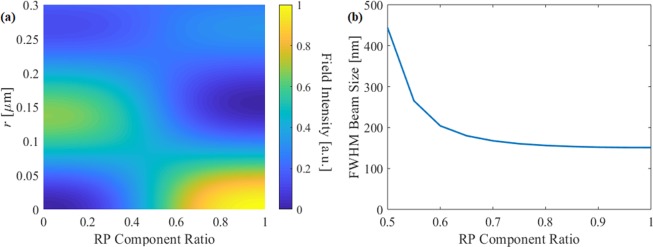
(**a**) Field intensity along the radial axis at the focal plane as a function of polarization ratio. (**b**) FWHM beam size at the focal plane as a function of polarization ratio.

In Fig. 1 we see very significant differences between the cases where AP and RP light illuminate the SOL, as would be expected. Figure 1 shows the AP component generates a donut-like field at the focal plane. The AP light retains its azimuthal symmetry as it is focused down, therefore all the fields at the center are cancelled due to cylindrical symmetry, and so the intensity is zero on the *z* axis. The peak of the donut-shaped field is at a radius *r* = 138 nm, which has the effect of enlarging the LP-SR focusing point when using mixed illumination. The RP-illuminated focal spot is tightly confined (full width half max, FWHM = 151 nm) and (by symmetry) contains only LP components.

To quantify the efficiency of focusing, we calculate the total energy in the focal spot for both RP and AP by integrating the intensity in the region inside the FWHM of the RP SR focus. We then calculate the polarization extinction ratio (PER) as the ratio of the energy between RP input and AP input, and the calculated PER of the SOL without sub-slits was 5.29 dB.

The field intensity at the focal plane and the beam width are both shown in Fig. 2 as a function of the mode ratio. From Fig. 2(a), we see that when the AP component exceeds 50%, the maximum is shifted from the center axis, where the lens no longer forms a well-defined focus at the focal plane. In addition, Fig. 2(b) shows that the beam width increases rapidly as the portion of AP component increases. The results show that if 40% of AP component is present, beam size increases to 200 nm, which is no longer sub-diffraction limited. Moreover, the AP component has azimuthal (*ϕ*) polarization at the focal plane, which hinders the pure LP focusing. Therefore, maintaining pure RP at the SOL is essential for high quality LP-SR focusing.

**Figure 3:**
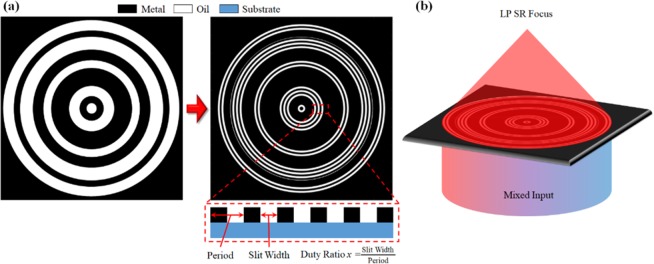
(**a**) Schematic of RP SOL. (**b**) Polarization-selective behaviours of the RP-SOL.

### SOL with subwavelength rings

To apply the annular slit arrays to our SOL, we substitute the transparent region of the SOL with subwavelength rings, which act as a wire-grid polarizer. A schematic of the RP-SOL is shown in Fig. 3. The RP-SOL will behave as a metallic layer for AP input components (and therefore block them) and will be transparent to the RP input components, (and therefore act as an SOL), overall forming an LP-SR focal point. The main design parameters of the subwavelength ring slits are: thickness of the metallic layer, the period of the slit, and the duty ratio between the metallic region and dielectric region, as depicted in Fig. 3(a).

**Figure 4:**
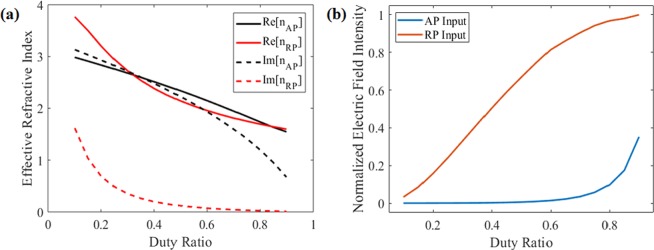
(**a**) Effective refractive index calculated via EMT. (**b**) Electric field intensity at the focal position for RP and AP input in terms of duty ratio.

To optimize the transmission and PER of the annular slits, we first optimize the thickness, duty ratio, and slit width without considering the SOL super-structure. Each parameter has trade-offs. In principal, the period does not affect the transmission in EMT, but high period structures (>0.5 λ) caused the EMT approximation to fail as they cannot be considered as a single material. As the slit period increases, its polarization selectivity reduces and it permits AP light to be transmitted. There is another problem for long periods: as (in general) not all openings of SOL are exact multiples of the period, sub-wavelength annular rings positioned at the edge of the SOL opening can have uneven width compared to the other rings. These slits then have a phase propagation constant slightly different from that of the standard-width slits, which can disturb the transmitted field. The larger the period, the higher the weight of the slit on this edge, which in turn leads to a decrease in the SOL efficiency. However, a short period causes makes the SAS-SOL harder to manufacture and may reduce the durability of the lens.

The duty ratio determines the effective refractive index of the annular slit arrays. A higher proportion of dielectric material reduces the loss, but also reduces the polarization selectivity (allowing more AP light through). A higher metallic proportion results in high imaginary component, therefore it efficiently blocks the AP component but also results in higher loss for RP inputs. The theoretically calculated effective indices for RP and AP input are shown in Fig. 4(a). The imaginary part of the index for each polarization component shows the effect of the duty ratio. The field intensity at the focal point for each polarization in terms of duty ratio are shown in Fig. 4(b). One can observe that the AP component is nearly eliminated for duty ratio <0.4.

**Figure 5:**
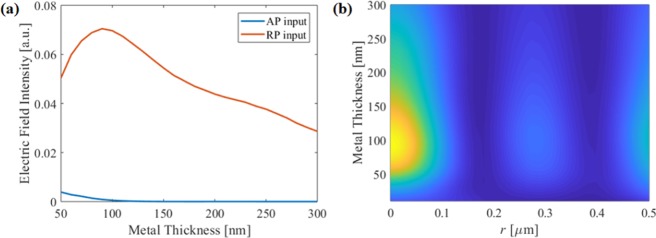
(**a**) Electric field intensity at the focal position for RP and AP input as a function of duty ratio. (**b**) Electric intensity distribution as a function of metal thickness for RP input.

In terms of thickness, as the AP response of the nano sub-slits is similar to metal, and so the transmission of the AP component decays exponentially as a function of metal thickness. For the RP response, the transmission has a peak at thickness of ~100 nm, and slowly decays because the effective medium acts as a lossy dielectric. The electric field intensity at the desired hotspot as a function of medium thickness is shown in Fig. 5. The electric field intensity decays exponentially for AP input, and the RP input shows a maximum at a thickness of around 90 nm. Note that for thin metal thickness (<30 nm) the incident light is not properly blocked, so the SOL fails to focus even for RP incident light.

**Figure 6:**
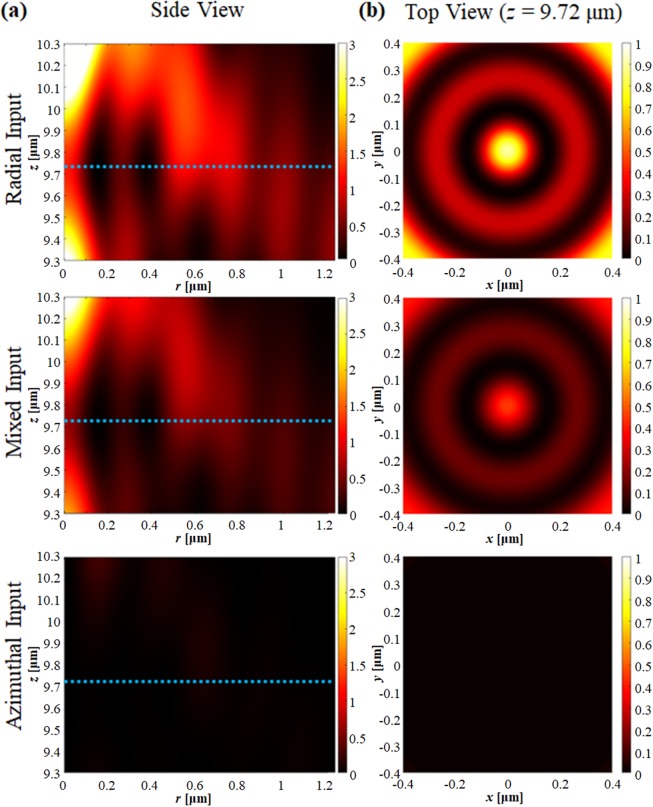
(**a**) Side view and (**b**) top view of the electric field intensity when RP, mixed, and AP light illuminates the SAS-SOL.

We find the intensity of SR focus is optimized at a duty ratio of 0.71, period of 75 nm, and metal thickness of 92 nm. The focal spots for a SAS-SOL with these optimised parameters for RP, AP and mixed inputs are shown in Fig. 6. It is clear that the AP component is strongly filtered out, especially around the SR spot. The field distribution for RP input is almost identical to that for a conventional SOL lens with FWHM = 152 nm (0.24 λ_0_ and 0.36 λ_oil_), and the efficiency shows only 0.342 dB of degradation, and the PER is 21.8 dB, giving 16.5 dB of improvement.

### Robustness and general applicability

Having shown the design process and specific results above, we now must demonstrate that SAS-SOLs designed in this way can feasibly be fabricated, and that the method is not limited to that particular design, but can be extended to other designs from the literature. First, we have simulated our designed structure with relatively large marginal errors of ±10 in both duty cycle and metal thickness. We do not vary the period this is stable in most fabrication processes. The results showed the FWHM was 153.8 nm, which is only 1.18% larger than the optimized case. The PER was more than 18.83 dB, where it was 21.8 dB originally. The output intensity fluctuated within a range of 10.69% (0.49 dB). Note that when the duty ratio was smaller (i.e. smaller open slits), the PER was increased, and when the duty ratio was larger (i.e. larger slits), the output power increased. Thus we show that even allowing for 10% marginal errors in fabrication the SAS-SOL can effectively generate a pure radially-polarised superoscillatory focus.

**Figure 7:**
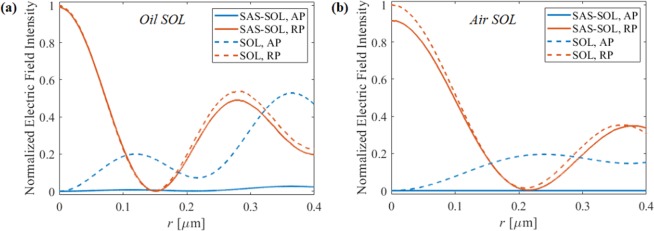
Electric field intensity distribution at the focal plane of the SAS-SOL (solid lines) and SOL without sub-slits (dashed lines) with AP input (blue lines) and RP input (red lines). The focusing medium is (**a**) oil and (**b**) air.

In addition, we have applied our SAS approach to other published SOLs from ref. ^32^. We have chosen two designs, SOL_5_ and SOL_4_ in table 1 from ref. ^32^. For SOL_5_, we have used the same materials – focusing in oil and using chromium – hereinafter “oil SOL”. And for SOL_4_, the light was focused in air and the metal was selected as aluminium – hereinafter, “air SOL”. As the conditions were same for the oil SOL as for our original design, no other optimization was required. However, as the materials were changed for the air SOL, we have optimized the duty ratio, period, and metal thickness again. The optimal parameters for the aluminium SAS-SOL focusing light in air were found to be a duty ratio of 0.22, period of 75 nm, and metal thickness of 180 nm. The electric field intensity at the SOL focal plane is shown in Fig. 7 for both SOLs, where the solid lines are the results of SAS-SOL and dashed lines are the results without sub-slits (the original design of ref. ^32^). The blue lines and red lines are the results when AP and RP input illuminated the lens, respectively. In the case of the oil SOL, the FWHM was 0.23 λ_0_ (0.35 λ_oil_) in both cases, the intensity loss after sub-slits was only 0.19%, and the PER is increased from 9.8 dB to 23.6 dB. In the case of the air SOL, the FWHM of both lenses is 0.32 λ_0,_ the intensity loss after sub-slits was 7.2%, and the PER has been increased from 12.1 dB to 105.5 dB. There results show that our SAS approach is robustly applicable to various different SOLs.

## Discussion

We have demonstrated a new SAS-SOL which generates, with a single optical component, a purely longitudinally-polarized subwavelength hotspot. Annular subwavelength slits are manufactured in the openings of conventional SOL structure, which selectively transmit RP light. This RP light is converted to LP by the focusing action of the SOL. Subwavelength slits were optimized using EMT theory, and numerical calculations were performed to characterize the SAS-SOL. 21.8 dB of mode purity has been calculated at the SR focusing spot with only 0.342 dB degradation of efficiency compared to the SOL without subwavelength rings. We have also shown the robustness of our design to fabrication errors and demonstrated the generality of the idea to other SOLs from the literature. The SAS-SOL simplifies the generation of LP-SR hotspots by substantially reducing alignment complexity and allowing monolithic integrated components. The SAS-SOL will find wide applications in fields where nano-confinement of light is needed: including imaging, data storage and nano-manufacturing with light.

## Methods

It is well known that sub-wavelength linear metallic structures transmit electric fields polarized perpendicular to the slit direction but block parallel polarization components^33^. It has recently been reported that arrays of annular slits selectively transmit RP light and block AP light, and this has been applied to binary Fresnel zone plates^30^. The effective refractive index of the metallic slit arrays for radial and azimuthal inputs calculated via EMT shows that metallic annular slit arrays respond as a ‘dielectric’ to RP and ‘metallic’ to AP inputs:

$$\begin{array}{rcl}{n}_{{\rm{eff}},{\rm{RP}}} & = & \frac{1}{\sqrt{\frac{f}{{n}_{1}^{2}}+\frac{1-f}{{n}_{2}^{2}}}}\\ {n}_{{\rm{eff}},{\rm{AP}}} & = & \sqrt{f{n}_{1}^{2}+(1-f){n}_{2}^{2}}\end{array}$$

where *n*_eff_ is the effective refractive index for each input component, *f* is the fraction of the dielectric, and *n*_1_ and *n*_2_ are the refractive index of the dielectric and metal, respectively. Our recent work also presents the effect of annular nanoslits, which works as a cylindrical symmetric polarizer^24^.

We perform FEM numerical simulations via COMSOL to analyze the characteristics of SOL for RP, AP, and combined input. Note that circular optical waveguides have second order degenerate modes with both RP and AP components^26^. The SOL used on the simulation is the lens from ref. ^12^. Chromium^34^ has been selected as the metallic material and the lens is covered with immersion oil (*n* = 1.515), in which the focus is formed. The material parameters of aluminium were taken from ref. ^35^. The expected SR focal length of the lens is 9.8 µm.
